# MYB82 functions in regulation of trichome development in *Arabidopsis*

**DOI:** 10.1093/jxb/eru179

**Published:** 2014-05-06

**Authors:** Gang Liang, Hua He, Yang Li, Qin Ai, Diqiu Yu

**Affiliations:** ^1^Key Laboratory of Tropical Forest Ecology, Xishuangbanna Tropical Botanical Garden, Chinese Academy of Sciences, Kunming, Yunnan 650223, China; ^2^University of Chinese Academy of Sciences, Beijing 100049, China

**Keywords:** MYB82, GL1, GL3, trichome, *Arabidopsis*.

## Abstract

Trichome initiation and patterning are controlled by the TTG1–bHLH–MYB regulatory complex. Several MYB transcription factors have been determined to function in trichome development via incorporation into this complex. This study examined the role of MYB82, an R2R3-MYB transcription factor, in *Arabidopsis* trichome development. MYB82 was revealed to be a nuclear-localized transcription activator. Suppression of MYB82 function by fusion with a dominant repression domain (SRDX) resulted in glabrous leaves, as did overexpression of N-terminal-truncated MYB82. Overexpression of *MYB82* genomic sequence, but not its cDNA sequence, led to reduced trichome numbers. Further investigation indicated that at least one of the two introns in *MYB82* is essential to the protein’s trichome developmental function. An MYB-binding box was identified in the third exon of *MYB82*, which was inferred to be crucial for *MYB82* function because the mutation of this box interfered with the ability of *MYB82* to rescue the *gl1* mutant. Protein interaction analysis revealed that MYB82 physically interacts with GLABRA3 (GL3). In addition, MYB82 and GL1 can form homodimers and heterodimers at R2R3-MYB domains, which may explain why their overexpression reduces trichome numbers. These results demonstrate the functional diversification of MYB82 and GL1 in trichome development.

## Introduction

Trichomes are single-celled epidermal hairs that help protect plants against herbivores, transpirational water loss, and UV irradiation (Marricio and Rausher, 1997; Serna and Martin, 2006). In *Arabidopsis*, trichomes exist on most aerial plant parts, including rosette leaves, stems, cauline leaves, and sepals, but not on hypocotyls and cotyledons. Trichome morphology and density vary among different organs. Trichomes on rosette leaves contain three to four branches, whereas cauline and stem trichomes are less branched or unbranched, respectively. Trichome density on the abaxial side of leaves increases with plant transition from vegetative to reproductive growth (Telfer *et al.*, 1997).

Trichome formation is controlled by programmed cell determination. The initiation and formation of trichomes has been extensively studied. The trichome developmental process is strictly controlled by transcription factors, which recognize specific DNA motifs in gene regulatory regions to activate or repress transcription, possibly through interaction with other proteins. Trichome initiation is regulated by a network under the control of a WD40–bHLH–MYB complex. In *Arabidopsis*, TRANSPARENT TESTA GLABRA1 (TTG1), a WD40 repeat protein, regulates trichome differentiation. Loss of function of TTG1 can also result in a lack of trichomes (Walker *et al.*, 1999). GL3 and EGL3 are two functionally redundant bHLH transcription factors, both of which regulate trichome initiation and act as a bridge to mediate the interaction between MYB and TTG1 (Payne *et al.*, 2000; Zhang *et al.*, 2003). The R2R3-MYB transcription factor GL1 is necessary for trichome initiation, and its mutation causes glabrous leaves (Oppenheimer *et al.*, 1991). MYB23 is functionally equivalent to GL1 during trichome initiation, and they redundantly regulate trichome initiation at leaf edges (Kirik *et al.*, 2005). In contrast to R2R3-MYB proteins, single-repeat R3-MYB proteins act as negative regulators of trichome development. These R3-MYB proteins consist of seven members: CAPRICE (CPC), TRIPTYCHON (TRY), ENHANCER OF TRY AND CPC 1 (ETC1), ETC2, ETC3, TRICHOMELESS1 (TCL1), and TCL2. CPC, TRY, ETC1, ETC2, and ETC3 function redundantly in trichome development (Esch *et al.*, 2004; Kirik *et al*., 2004a,b; Schellmann *et al.*, 2007; Wester *et al.*, 2009). TCL1 plays an important role in trichome formation on stems and pedicels (Wang *et al.*, 2007), and TCL2 functions redundantly with TCL1 in controlling trichome formation on inflorescences (Gan *et al.*, 2011). It has been suggested that single-repeat R3-MYB proteins negatively regulate trichome development by competing with GL1 for binding to GL3/EGL3, resulting in disruption of (R2R3-MYB)–bHLH–TTG1 complexes (Payne *et al.*, 2000; Bernhardt *et al.*, 2003; Esch *et al.*, 2003; Ishida *et al.*, 2008).

The WD40–bHLH–MYB complex regulates the expression of downstream genes to control trichome development. GLABRA2 (GL2), a homeodomain (HD-Zip) transcription factor, is required for trichome morphogenesis. The *gl2* mutant produces abnormal trichomes (i.e. most of the trichomes do not expand, and possess only a single branch; Rerie *et al.*, 1994; Masucci *et al.*, 1996; Fyvie *et al.*, 2000; Ohashi *et al.*, 2002). TRANSPARENT TESTA GLABRA2 (TTG2), a WRKY transcription factor, shares functions with GLABRA2 in controlling trichome outgrowth. Mutation in *TTG2* causes unbranched trichomes (Johnson *et al.*, 2002). *GL2* is directly regulated by GL1, GL3, and EGL3 (Morohashi *et al.*, 2007; Zhao *et al.*, 2008), and *TTG2* is a direct target of GL1 (Ishida *et al.*, 2007; Zhao *et al.*, 2008).

This study focused on the function of MYB82 in trichome development and demonstrated that MYB82 functions in the plant cell nucleus as a positive regulator. Although MYB82 loss of function did not disrupt trichome development, MYB82 overexpression led to abnormal trichome development. On the other hand, overexpression of MYB82-SRDX (fused with a dominant repression domain) or an N-terminal R2R3-MYB domain caused glabrous leaves. *MYB82* driven by the *GL1* promoter was able to rescue the glabrous phenotypes of the *gl1* mutant, suggesting that the MYB82 protein is functionally equivalent to the GL1 protein. The *MYB82* gene contains two introns, at least one of which was found to be crucial for MYB82 regulation of trichome development. The third exon of *MYB82* contains a perfect MYB-binding box that was demonstrated to be also necessary for MYB82 function. Similar to GL1, MYB82 was able to interact with GL3, suggesting that MYB82 is incorporated into the WD40–bHLH–MYB complex to participate in the regulation of trichome development. In addition, this study revealed that MYB82 proteins can form homodimers or heterodimers with GL1 proteins.

## Materials and methods

### Plant materials

*Arabidopsis* ecotype Col-0 and its *gl1* mutant were used for experiments. Plants were grown at 23 °C under a 16/8h light/dark cycle or a 8/16 light/dark cycle.

### Subcellular localization

Full-length cDNA of *MYB82* was fused with that of the eGFP protein in a frame downstream of the 35S promoter in a pOCA30a binary vector. The resulting 35S:MYB82-eGFP plasmid was transformed into *Agrobacterium tumefaciens* strain EHA105. The transformed *Agrobacterium* was incubated, harvested, and resuspended in infiltration buffer (0.2mM acetosyringone, 10mM MgCl_2_, and 10mM MES, pH 5.6) and then infiltrated into *Nicotiana benthamiana* leaves. After infiltration, plants were incubated at 24 °C for 48h before observation. DAPI staining was used for visualization of cell nuclear DNA. GFP and DAPI fluorescence were observed under a confocal laser scanning microscope (Olympus, Tokyo, Japan).

### Real-time quantitative RT-PCR

Total RNA (1 μg), extracted using Trizol reagent (Invitrogen), was used for oligo(dT)18-primed cDNA synthesis according to the reverse transcription protocol (Fermentas). The resulting cDNA was subjected to real-time quantitative RT-PCR using a SYBR Premix Ex Taq kit (Takara) on a Roche LightCycler 480 real-time PCR machine. For each reported result, at least three independent biological samples were subjected to a minimum of three technical replicates. The results were normalized using the internal control *ACTIN2*. The primers for quantitative RT-PCR are listed in Supplementary Table S2 (available at *JXB* online).

### Plasmid construction

For the 35S:MYB82-eGFP plasmid, MYB82 cDNA was fused in-frame to the 5′-terminal of eGFP driven by the CaMV 35S promoter in a pOCA30a binary vector. For the 35S:MYB82(/GL1)-SRDX plasmid, the *MYB82/GL1* genomic sequence was fused with the minimal repression domain (DLELRL). *GL1* regulatory sequences used in these experiments were described previously (Lee and Schiefelbein, 2001) and included a 1.4-kb 5′-fragment and a 1.8-kb 3′-fragment. A 5×GAL4+35S minimal promoter sequence was synthesized and inserted into an upstream GUS gene in a binary vector. GAL4 DNA-binding domain (BD) and GAL4 activation domain (AD; AD domain containing nuclear-localized signal from SV40) sequences were amplified from pGBKT7 and pGADT7 plasmids, respectively. The primer sequences are listed in Supplementary Table S2. Plasmid construction details are available upon request.

### GUS reporter analysis

The putative promoters of *MYB82* were amplified from genomic DNA using primers Pro-MYB82-F and Pro-MYB82-R (Supplementary Table S2). The fused *Pro*_*myb82*_ *-GUS* was cloned into the pOCA28 vector. Transgenic plants were subjected to GUS staining as described by He *et al.* (2014).

### Yeast two-hybrid assay

Fusions with the GAL4 activation domain and the GAL4 DNA-binding domain were performed in the pGBKT7 and pGADT7 plasmids. Full lengths or fragments of GL3, MYB82, and GL1 were individually cloned into pGBKT7 or pGADT7 plasmids. Growth was determined as described in the Yeast Two-Hybrid System user manual (Clontech).

### Bimolecular fluorescence complementation assay

Full-length coding sequences of *MYB82* and *GL3* were cloned into binary N-terminal fragment of yellow fluorescent protein (nYFP)and C-terminal fragment of yellow fluorescent protein (cYFP) vectors, respectively. *Agrobacterium* strains transformed with indicated nYFP or cYFP vectors were incubated, harvested, and resuspended in infiltration buffer (0.2mM acetosyringone, 10mM MgCl_2_, and 10mM MES, pH 5.6) to identical concentrations (*A*_600_=0.5). Equal volumes of an *Agrobacterium* culture containing nYFP (*A*_600_=0.5) and cYFP (*A*_600_=0.5) were mixed before infiltration into *N. benthamiana* leaves. After infiltration, plants were incubated at 24 °C for 48h before observation.

## Results

### MYB82 is a nuclear-localized transcription activation factor

**Fig. 1.**
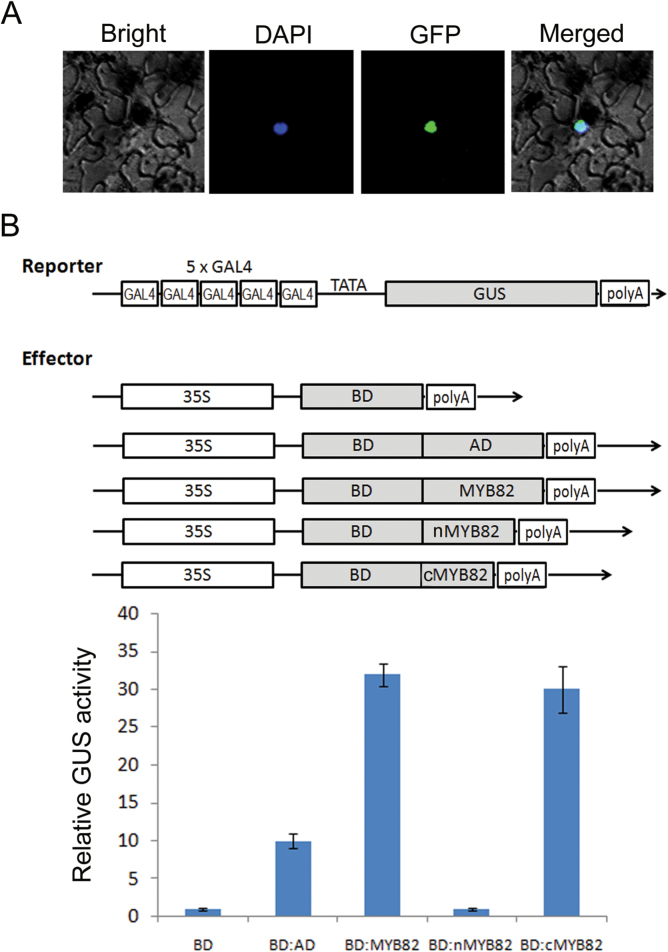
MYB82 is a nuclear-localized transcription activation factor. (A) Confocal images showing the subcellular distribution of eGFP-tagged MYB82 fusion proteins in transiently transformed tobacco cells; *Nicotiana benthamiana* plants were infiltrated with *Agrobacterium tumefaciens* cells harbouring 35S:MYB82-eGFP plasmids. (B) Transcription activation of MYB82 tethered to DNA through Gal4 BD (DB:MYB82); reporter and effector were transiently coexpressed in tobacco cells.

To identify the subcellular localization of MYB82, its coding region was fused with an *ENHANCED GREEN FLUORESCENT PROTEIN* (*eGFP*) reporter gene under the control of the cauliflower mosaic virus (CaMV) 35S promoter. Tobacco epidermal cells transformed to transiently express MYB82-eGFP showed considerable fluorescent signal in cell nuclei, with no signal observed in other compartments (Fig. 1A). This result suggested that MYB82 is a nuclear-localized transcription factor.

MYB transcription factors function as transcription activators or repressors (Dubos *et al.*, 2010). To clarify whether MYB82 is a transcription activator or a transcription repressor, this work performed transient transcription assays in tobacco leaves. The reporter plasmid contained five tandem copies of the GAL4-binding site upstream of the *GUS* reporter gene (Fig. 1B). Full-length MYB82 was fused to the GAL4 BD downstream of the 35S promoter as an effector plasmid (Fig. 1B). The GAL4 AD was fused to BD to generate GAL4-BD:AD as a positive effector. Compared with GAL4-BD, GAL4-BD:AD significantly stimulated expression of GUS. When GAL4-BD:MYB82 was coexpressed with the reporter, GUS activities were higher than those observed from GAL4-BD:AD coexpression with the reporter (Fig. 1B). These data demonstrated that MYB82 was able to activate transcription via its intrinsic activation domain.

To further confirm which domain functions as a transcription activation domain, the N-terminal R2R3-MYB domain (nMYB82) and C-terminal domain (cMYB82) were separately fused to GAL4-BD. Coexpression results indicated that the C-terminal domain was responsible for the transcription activation function. Taken together, these data suggested that MYB82 is a nuclear-localized transcription activation factor.

### Function repression of *MYB82* resulted in glabrous leaves

**Fig. 2.**
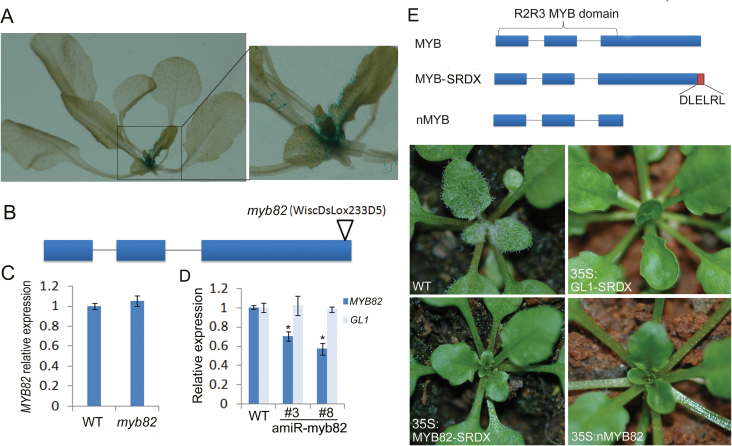
Function repression of MYB82 caused a reduction in leaf trichome number. (A) GUS staining of *Pro*_*myb82*_ *:GUS* reporter line. (B) Location of T-DNA in the *MYB82* gene; bars and lines indicate exons and introns, respectively; the triangle indicates the location of the T-DNA. (C and D) Transcript abundance of *MYB82* in the *myb82* mutant (C) and *amiR-myb82* transformants (D); transcript abundance of GL1 is shown as a negative control; #3 and #8 correspond to two independent amiR-myb82 transformants. (E) Phenotypes of transformants with altered MYB proteins; red bar indicates the SRDX domain (DLELRL), MYB-SRDX indicates the MYB protein fused with an SRDX domain, and nMYB indicates the MYB protein containing only the R2R3 MYB domain.

Although the functions of *MYB82* are unclear, its protein sequence is highly similar to GL1 (Supplementary Fig. S1), an R2R3-MYB subgroup-15 member (Dubos *et al.*, 2010) and a key regulator of trichome initiation (Oppenheimer *et al.*, 1991). This work speculated that *MYB82* might be involved in regulation of trichome development. To confirm this hypothesis, first the expression pattern of MYB82 was determined. The upstream sequence of MYB82 was used to drive the GUS reporter gene (*Pro*_*MYB82*_ *:GUS*), which allowed transcription regulation of *MYB82* to be monitored. GUS staining suggested that *Pro*_*MYB82*_ *:GUS* was mainly expressed in the trichomes of new leaves (Fig. 2A), which implied that MYB82 might function in the initiation of trichomes. This work then obtained a homozygous T-DNA insertion line for *MYB82*, which was generated by the deletion of seven C-terminal amino acids (Fig. 2B). Expression analysis revealed that the abundance of truncated transcripts in the mutant was similar to the level of full-length transcripts present in the wild type (Fig. 2C). Moreover, this mutant was not phenotypically different from wild-type plants. These results suggested that the MYB82 3′-terminal deletion may not affect this protein’s function. Therefore, this work used an artificial miRNA approach (Liang *et al.*, 2012) to suppress *MYB82* expression. An *amiR-myb82* sequence was designed using WMD3 (http://wmd3.weigelworld.org), with an *Ath-miR395a* backbone used to drive its expression (Liang *et al.*, 2012). The *amiR-myb82* precursor was inserted into the downstream of the 35S promoter in a binary vector. Twenty transgenic plants were further analysed for *MYB82* transcript levels (Supplementary Fig. S1). As expected, *MYB82* mRNA abundance was decreased in *amiR-myb82* transgenic plants, with *GL1* mRNA unchanged (Fig. 2D); however, no obvious phenotypic difference was observed between *amiR-myb82* and wild-type plants, implying that MYB82 may function redundantly with its closely homologous genes.

Based on the fact that fusion of a dominant repression domain to a transcriptional activator can convert the latter into a strong repressor by repressing target gene expression (Hiratsu *et al.*, 2003), this work employed a dominant repression approach to investigate MYB82 function. Transgenic *Arabidopsis* plants (*35S:MYB82-SRDX*) expressing MYB82 fused with the dominant EAR repression domain (Hiratsu *et al.*, 2004) were generated. The resulting *35S:MYB82-SRDX* transgenic plants produced nearly glabrous leaves with a few trichomes in leaf margins (Fig. 2E), reminiscent of the phenotypes exhibited by the trichome defective mutant *gl1*. As a positive control, *35S:GL1-SRDX* transgenic plants were also generated; as expected, *35S:GL1-SRDX* transgenic plants produced completely glabrous leaves.

The MYB82 protein consists of one N-terminal R2R3-MYB DNA-binding domain and one C-terminal activation domain. This work hypothesized that overexpression of the N-terminal DNA-binding domain would confer a dominant negative effect by preventing endogenous MYB82 or homologous proteins from associating with their target DNA motif. When nMYB82 with a deletion of the C-terminal domain was constitutively expressed (*35S:nMYB82*), trichome initiation completely failed, even in leaf margins.

*GL2*, a downstream target gene of WD40–bHLH–MYB complex, is expressed in the trichome and required for trichome development. This work investigated whether these transgenic plants with less trichomes have low *GL2* expression levels. As expected, the trichome number agreed well with *GL2* transcript abundance in these transgenic plants (Supplementary Fig. S2). Taken together, MYB82 positively regulates trichome development.

### *MYB82* introns affected its function

**Fig. 3.**
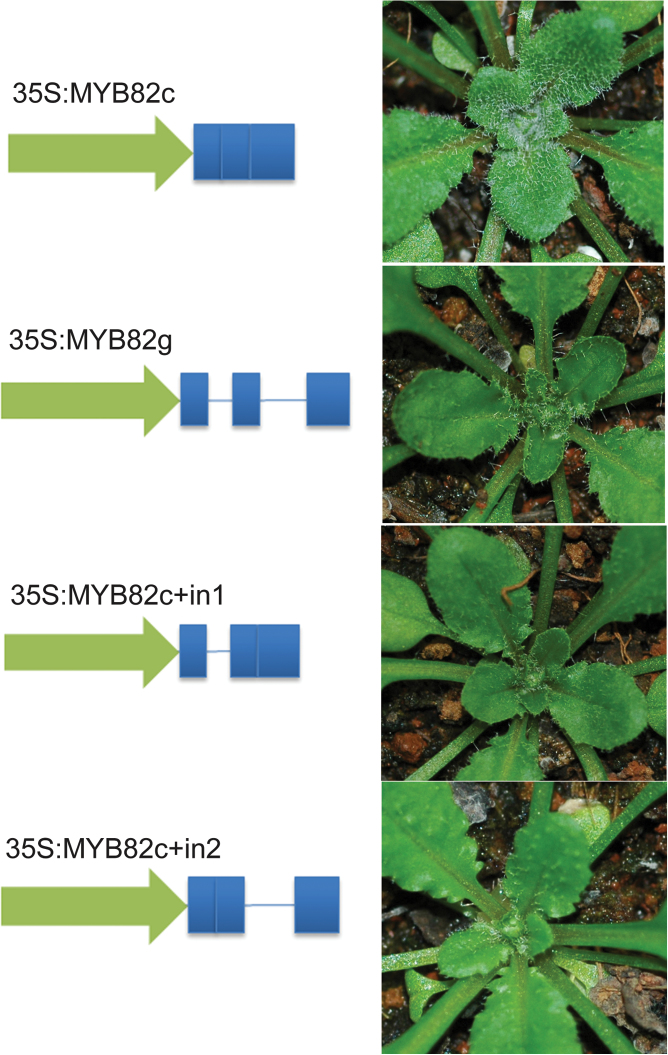
Effect of *MYB82* introns on trichome development. Schematic presentations of constructs are depicted on the left; arrows indicate the 35S promoter; bars and lines indicate *MYB82* exons and introns, respectively. Wild-type plants were used for transformation. Representative transformants are shown.

Although GL1 positively regulates trichome initiation, *GL1* overexpression reduces trichome number (Larkin *et al.*, 1994). To investigate the effects of constitutive and ectopic expression of *MYB82*, *MYB82* cDNA was inserted downstream of the 35S promoter in a binary vector. The *35S:MYB82c* construct was transformed into wild-type *Arabidopsis* plants. Unexpectedly, no visible trichome-defective phenotype (Fig. 3) was observed in any of the 37 *35S:MYB82c* transformants although most of them contained high levels of *MYB82* transcripts (Supplementary Table S1). It has been suggested that the second introns of *GL1* and *GaMYB2* contain an MYB-binding box required for gene function (Wang *et al.*, 2004); however, the current work was unable to find a similar MYB-binding box in the two introns of *MYB82*. Considering the possibility that unknown *cis*-elements are present in the introns, transgenic plants constitutively expressing the *MYB82* genomic sequence (*35S:MYB82g*) were constructed (Fig. 3). Among the 30 *35S:MYB82g* transformants, 70% surprisingly showed reduced trichome numbers, with the remaining 30% exhibiting no difference compared with wild-type plants. This result implied that the introns of *MYB82* play important roles in gene function. To determine which intron was involved in gene functional regulation, two constructs (*35S:MYB82c+in1* and *35S:MYB82c+in2*), each containing *MYB82*c and one of the two introns, were produced (Fig. 3). The phenotypes of *35S:MYB82c+in1* and *35S:MYB82c+in2* transgenic plants were similar, both displaying reduced trichome numbers. It thus appears that at least one intron is involved in the function of *MYB82* in trichome development.

### Both *pGL1:MYB82g* and *pGL1:MYB82c* could rescue *gl1* mutants

**Fig. 4.**
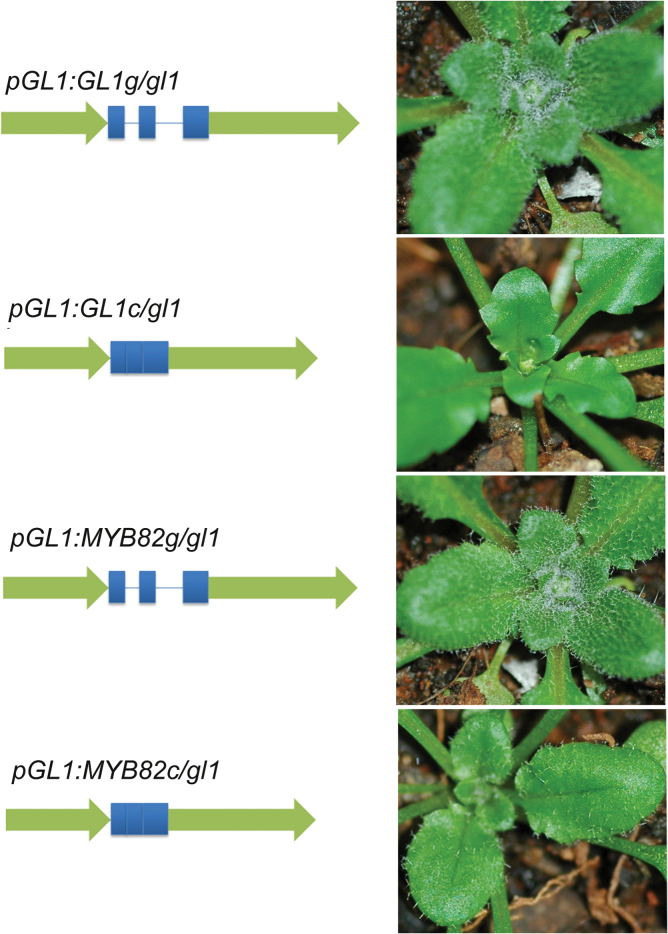
Rescue of the *gl1* mutant by MYB82. Schematic presentations of constructs are depicted on the left; arrows indicate the GL1 promoter; bars and lines indicate *MYB82* exons and introns, respectively. *gl1* mutant plants were used for transformation. Representative transformants are shown.

Analysis of MYB82 loss- and gain-of-function suggested that MYB82 could mediate *Arabidopsis* trichome development. Given that MYB82 displayed a function similar to GL1, this work investigated whether the MBY82 protein was able to complement the trichome defect of the *gl1* mutant. Because 1.4-kb upstream and 1.8-kb downstream regions of the *GL1* gene are necessary for its appropriate expression (Lee and Schiefelbein, 2001), these two regions were used for the *GL1* promoter (Fig. 4). As a positive control, the *GL1* promoter was used to drive *GL1c* or *GL1g* in the *gl1* mutant (Fig. 4). In accordance with a previous report (Wang *et al.*, 2004), *pGL1:GLg*, but not *pGL:GLc*, was able to rescue the *gl1* mutant. *pGL1:MYB82g* and *pGL1:MYB82c* were constructed in a similar fashion. When these two constructs were separately introduced into the *gl1* mutant, *pGL1:MYB82g* completely complemented the mutant, with leaf trichome densities comparable to the wild type, whereas *pGL1:MYB82c* partially complemented the *gl1* mutant, with trichome densities lower than the wild type but higher than the *gl1* mutant (Fig. 4).

### The MYB-binding box in the third exon of *MYB82* was crucial for gene function

**Fig. 5.**
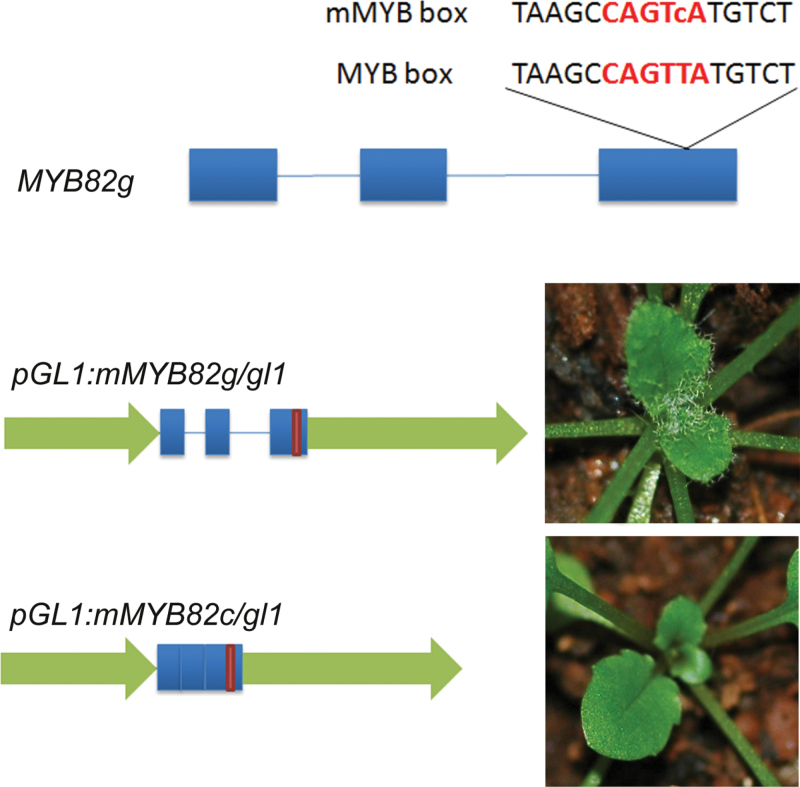
Effect of the MYB-binding box of *MYB82* on trichome development. Schematic presentations of constructs are depicted on the left; red letters indicate the wild-type and mutated MYB-binding box; arrows indicate the GL1 promoter; bars and lines indicate *MYB82* exons and introns, respectively; red bar indicates a mutated MYB-binding box.

Although *pGL1:MYB82c* promoted trichome initiation in the *gl1* mutant, *pGL1:GL1c* did not (Wang *et al.*, 2004). These results were confusing, because *35S:MYB82c* had no effect on trichome phenotypes. The first intron of the *GL1* gene contains an MYB-binding box required for *GL1* expression, which explains why *pGL1:GL1c* could not rescue the *gl1* mutant (Wang *et al.*, 2004). However, no MYB-binding boxes are present in *MYB82* introns. Speculating that an unidentified *cis*-element exists in exon sequences of *MYB82*, this work searched the complete *MYB82* genomic sequence and found a perfect MYB-binding box in the third exon (Fig. 5). In contrast, no MYB-binding boxes exist in *GL1* gene exons. This box is thus likely responsible for *MYB82* gene function. To confirm this hypothesis, this work changed the MYB-binding box sequence without altering the amino acid sequence to generate *mMBY82g* and *mMYB82c* (Fig. 5) and then used *pGL1:mMYB82g* and *pGL1:mMYB82c* in a complementation assay of the *gl1* mutant. As shown in Fig. 5, *pGL1:mMYB82g* partially complemented *gl1*, whereas *pGL1:mMYB82c* had no effect on *gl1* trichome initiation. These results suggested that the MYB-binding box in the third exon of *MYB82* is required for MYB82 function in trichome initiation.

### MYB82 was incorporated into the TTG1–bHLH–MYB complex

**Fig. 6.**
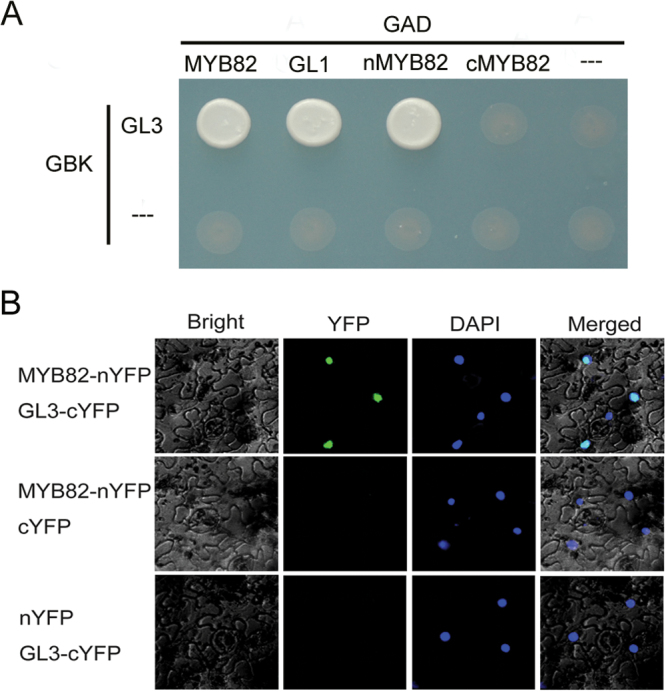
Interactor of the MYB82 protein. (A) Interaction of MYB82 with GL3 in yeast; interaction was indicated by the ability of cells to grow on synthetic dropout medium lacking Leu/Trp/His/Ade and containing 5mM 3AT; full-length MYB (MYB82 and GL1), N-terminal truncated MYB (nMYB), and C-terminal truncated MYB (cMYB) were cloned into pGBKT7; full-length GL3 was cloned into pGADT7. (B) Interaction of MYB82 with GL3 in plant cells; fluorescence was observed in nuclear compartments of *Nicotiana benthamiana* leaf epidermal cells, resulting from complementation of the N-terminal portion of YFP fused to MYB82 (MYB82-nYFP) with the C-terminal portion of YFP fused to GL3 (GL3-cYFP).

The conserved signature motif ([DE]Lx_2_[RK]x_3_Lx_6_Lx_3_R) of MYB proteins is crucial for their interaction with the bHLH protein GL3 that functions in trichome initiation and anthocyanin synthesis (Zimmermann *et al.*, 2004; Dubos *et al.*, 2010). This motif also exists in the MYB82 protein (Supplementary Fig. S3). To verify whether MYB82 interacts with GL3, the MYB82 full-length coding region was fused to the BD domain of pGBK-T7, and GL3 was fused to the AD domain of pGAD-T7. Yeast two-hybrid assays suggested that MYB82 interacted with GL3 (Fig. 6A). To determine which region of the MYB82 protein is responsible for this interaction, the N-terminal region containing the MYB domain and the C-terminal region were separately fused with the BD domain. The N-terminal region was found to be sufficient for MYB82 interaction with GL3 (Fig. 6A). To confirm whether this interaction also occurred in plant cells, bimolecular fluorescence complementation assays were employed (Fig. 6B). nYFP was ligated to MYB82 and cYFP was ligated to GL3. When MYB82-nYFP was transiently coexpressed with GL3-cYFP, strong YFP fluorescence was visible in epidermal cell nuclei of *N. benthamiana* leaves. No YFP fluorescence was detected in negative controls (i.e. MYB82-nYFP coexpressed with cYFP or nYFP coexpressed with GL3-cYFP; Fig. 6B). These results suggested that MYB82 physically interacts with GL3 in plant cell nuclei.

### MYB82 and GL1 formed homodimers and heterodimers

**Fig. 7.**
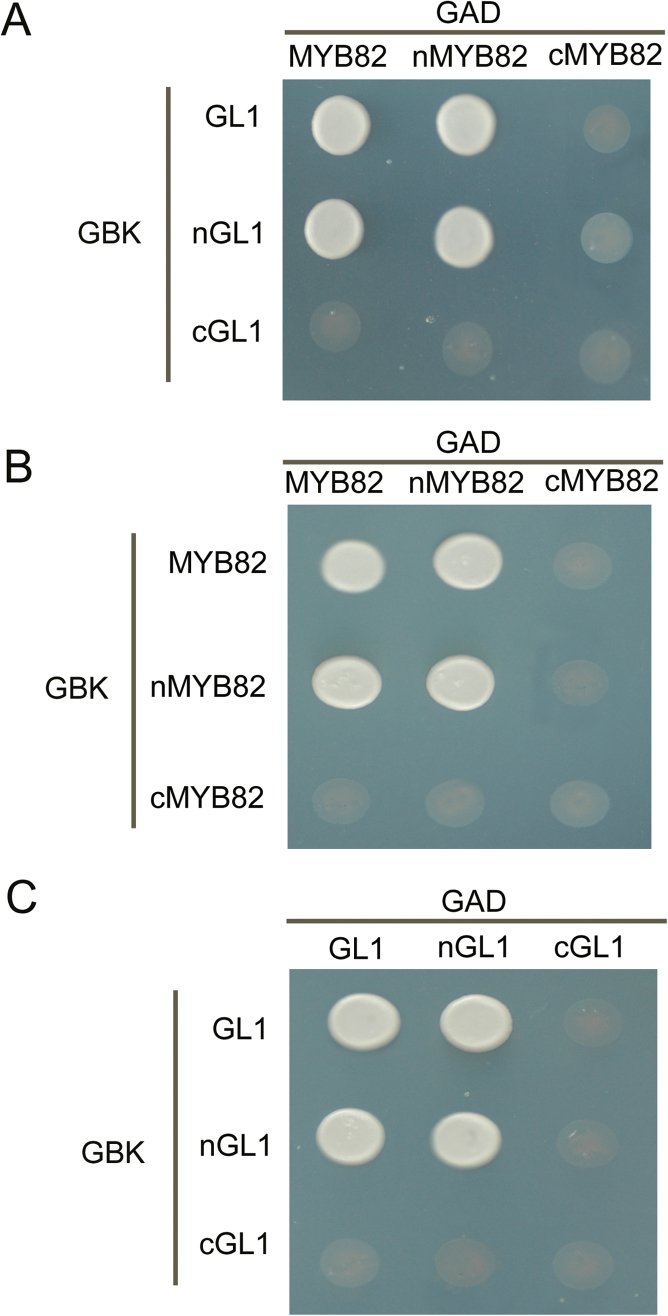
MYB82 and GL1 form homodimers and heterodimers. (A) MYB82 and GL1 form a heterodimer. (B) MYB82 displays homomeric interaction. (C) GL1 displays homomeric interaction. nMYB82 (nGL1) indicates an N-terminal truncated protein and cMYB82 (cGL1) indicates a C-terminal truncated protein. Interaction was indicated by the ability of cells to grow on synthetic dropout medium lacking Leu/Trp/His/Ade and containing 5mM 3AT.

Many transcription factors, including bHLH and bZIP transcription factors, function as homo- or heterodimers (Murre *et al.*, 1989; Ferre-D’Amare *et al.*, 1994; Vinson *et al.*, 2002). The Phosphate Starvation Response 1 (PHR1) protein containing a single MYB repeat has been shown to function as a dimer (Rubio *et al.*, 2001). Two closely related R2R3-MYB proteins, MYB21 and MYB24, were recently confirmed to form homo- and heterodimers (Song *et al.*, 2011). Given their similar protein sequences and functions, dimeric interaction likely exists between MYB82 and GL1. To verify this possibility, this work performed a yeast two-hybrid assay. As expected, both MYB82 and GL1 were able to form homodimers and heterodimers (Fig. 7). This work also determined the domain responsible for the interaction, finding that the N-terminal MYB domain is necessary and sufficient for dimer formation (Fig. 7A and B).

## Discussion

Most R2R3-MYB proteins have been characterized using genetic approaches and have been found to be involved in the control of plant-specific processes such as primary and secondary metabolism, cell fate and identity, developmental processes, and responses to biotic and abiotic stresses (Dubos *et al.*, 2010). A particularly interesting entity is the WD40–bHLH–MYB transcription complex, which has specific functions determined by different MYB proteins. For instance, the complex regulates anthocyanin biosynthesis in vegetable tissues when MYB75/90/113/114 is incorporated (Gonzalez *et al.*, 2008); when GL1/MYB23 is recruited, the complex regulates trichome initiation and branching (Kirik *et al.*, 2005; Zhao *et al.*, 2008). In roots, WER is present in the complex that controls root hair patterning (Lee and Schiefelbein, 1999). Here, it is suggested that MYB82 joins in the complex and positively regulates trichome development.

R2R3-MYB transcription factors have a modular structure consisting of an N-terminal DNA-binding domain (the MYB domain) and an activation or repression domain usually located at the C-terminus. MYB82 is an R2R3-MYB family member. Transient expression assays indicated that the MYB82 protein was localized in the plant cell nucleus (Fig. 1A) and contained an activation domain in its C-terminus (Fig. 1B). MYB82 showed high sequence similarity with GL1 (Supplementary Fig. S1), implying a role in trichome development. Similar to GL1 overexpression, elevated expression of MYB82 caused reduced trichome numbers. In addition, both *pGL1:MYB82g* and *pGL1:MYB82c* promoted trichome formation in the *gl1* mutant. These results demonstrate that MYB82 function is nearly equivalent to that of GL1. Because MYB82 is a transcription activation factor, the addition of the dominant repression domain (SRDX) to this protein almost completely suppressed trichome initiation; this result is similar to that obtained using *35S:GL1-SRDX* transgenic plants (Fig. 2B). Overexpression of N-terminal truncated protein, which may competitively suppress the association of paralogous MYB-related proteins with target gene *cis*-regulatory elements, caused completely glabrous leaves. This result suggests that MYB82 is able to promote expression of trichome development-associated genes.

In addition to protein function similarity to GL1, MYB82 also displayed functional divergence. This work found that *35S:MYB82g* and *35S:MYB82c* have different effects on trichome development. Further analysis suggested that at least one intron is required for MYB82 under the control of the 35S promoter to affect trichome development. The first intron of *GL1*, similarly to *WER* and *GaMYB2* genes, contains an MYB-binding box required for functional complementation of the *gl1* mutant (Wang *et al.*, 2004). No MYB-binding box was found in the introns of MYB82, although it is still possible that unidentified *cis*-regulatory elements are present. When *MYB82* driven by the *GL1* promoter was used to rescue the *gl1* mutant, *pGL1:MYB82g* showed complete function complementation of the *gl1* mutant, whereas *pGL1:MYB82c* only partially complemented the *gl1* mutant. In contrast, *pGL1:GL1g* can complement the *gl1* mutant, whereas *pGL1:GLc* has no contribution to mutant trichome initiation (Wang *et al.*, 2004). The phenotypic difference between *pGL1:MYB82c* and *pGL1:GL1c* in the *gl1* mutant background suggested that the exons of *MYB82* contain *cis*-regulatory elements that are absent from *GL1* exons. As expected, *MYB82* contains a perfect MYB-binding box in the third exon. Mutation of this MYB-binding box disrupted *pGL1:mMYB82c* complementation of the *gl1* mutant. Although *MYB82* is a paralogue of *GL1*, *MYB82* has evolved distinct *cis*-regulatory elements that directly affect its functions. Taken together, *MYB82* shows functional divergence from *GL1* in regard to trichome development.

R2R3-MYB subgroup 15 consists of three members: GL1, MYB23, and WER (Dubos *et al.*, 2010). MYB82 and GL1 protein sequences are highly similar to one another. Subgroup-15 members have been confirmed to be incorporated into the WD40–bHLH–MYB complex (Zimmermann *et al.*, 2004). The current work’s protein interaction analysis suggested that MYB82 also interacts with the bHLH transcription factor GL3, implying that MYB82 is involved in trichome development through participation in the WD40–bHLH–MYB transcription complex.

A contradictory phenomenon is the observation that overexpression of positive regulators of trichome development, GL1 and MYB23, as well as MYB82, often cause trichome number reductions (Larkin *et al.*, 1994; Kirik *et al.*, 2001). The current work revealed that MYB82 and GL1 can form homodimers or heterodimers at the N-terminal R2R3-MYB domain. It has been confirmed that, in the WD40–bHLH–MYB complex, MYB interacts with bHLH at its MYB domain (Grotewold *et al.*, 2000). It is therefore very likely that overexpression of the R2R3-MYB protein results in increased production of MYB homodimers or heterodimers, which may competitively lead to fewer WD40–bHLH–MYB complexes, and, consequently, to a reduction in trichome numbers.

## Supplementary material

Supplementary data are available at *JXB* online.

Supplementary Fig. S1. Expression levels of *MYB82* in *amiR-myb82* transgenic plants.

Supplementary Fig. S2. Expression levels of *GL2* in different transgenic plants.

Supplementary Fig. S3. MYB proteins containing the conserved signature motif ([DE]Lx_2_[RK]x_3_Lx_6_Lx_3_R).

Supplementary Table S1. Expression and phenotype analysis of transgenic plants.

Supplementary Table S2. Primer sequences.

Supplementary Data
