# Supplementary material for: MYB82 functions in regulation of trichome development in Arabidopsis
Source: J Exp Bot. 2014 May 6;65(12):3215–23. doi: 10.1093/jxb/eru179 (PMC4071844; doi:10.1093/jxb/eru179)
Supplement: Supplementary Data [file supp_65_12_3215__index.html]

MYB82 functions in regulation of trichome development in Arabidopsis — MYB82 functions in regulation of trichome development in Arabidopsis — Supplementary Data 

# MYB82 functions in regulation of trichome development in *Arabidopsis*

## Supplementary Data

Data files

**Files in this Data Supplement:**

- Supplementary Data - Supplementary Data
