# Supplementary material for: MYB82 functions in regulation of trichome development in Arabidopsis
Source: J Exp Bot. 2014 May 6;65(12):3215–23. doi: 10.1093/jxb/eru179 (PMC4071844; doi:10.1093/jxb/eru179)
Supplement: Supplementary Data [file supp_eru179_jexbot122069_file001.pdf]

MYB82 functions in regulation of trichome development in *Arabidopsis*

Gang Liang[1,3], Hua He[1,2,3], Yang Li[1,2,3], Qin Ai[1,2], Diqiu Yu[1]*

1 Key Laboratory of Tropical Forest Ecology, Xishuangbanna Tropical Botanical Garden, Chinese Academy of Sciences, Kunming, Yunnan 650223, China.

2 University of Chinese Academy of Sciences, Beijing 100049, China.

3 These authors contributed equally to this work.

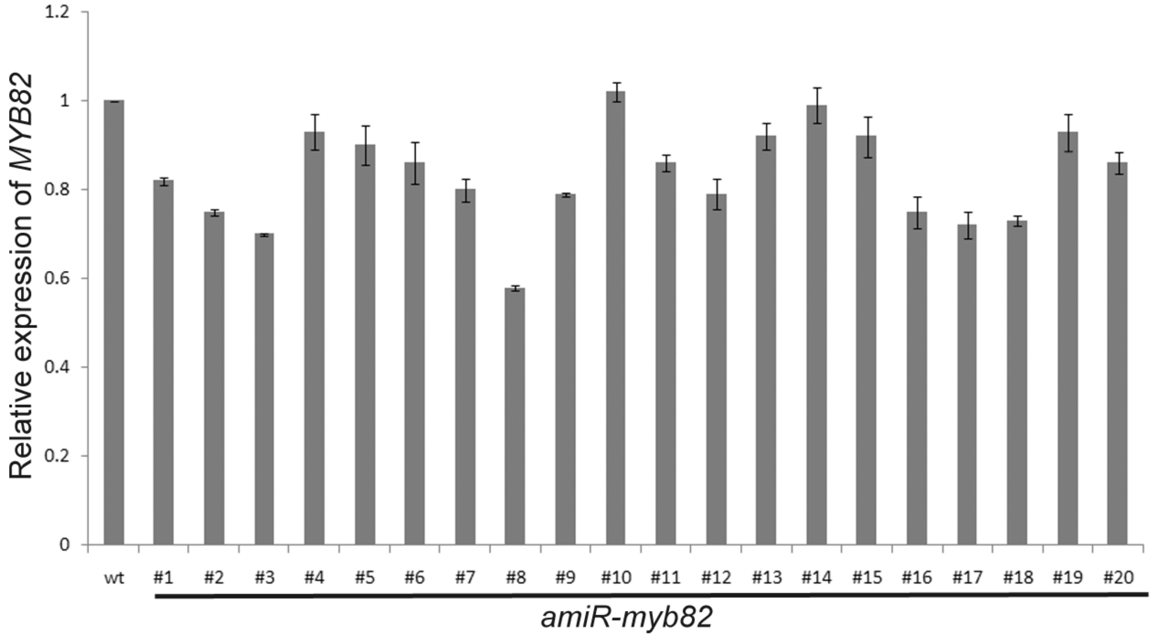

Figure S1. Expression levels of *MYB82* in amiR-myb82 transgenic plants.

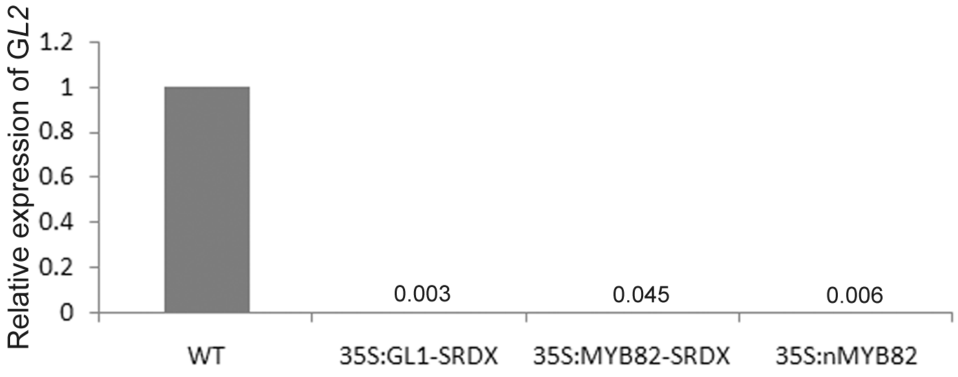

Figure S2. Expression levels of *GL2* in different transgenic plants.

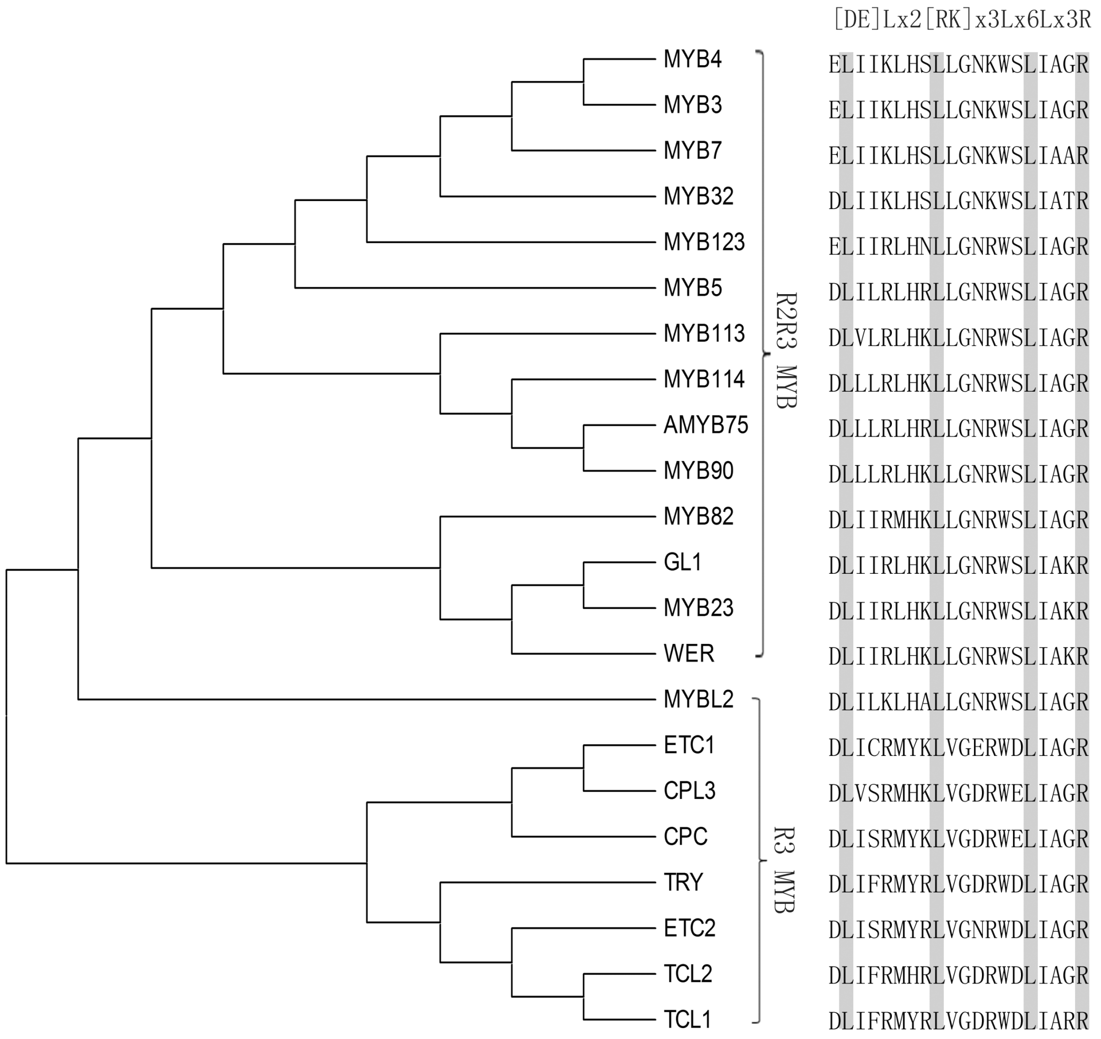

Figure S3. MYB proteins containing the conserved signature motif ([DE]Lx_2[RK]$x_3Lx_6Lx_3$R). Protein sequences were aligned by Clustal X2 software. The tree was inferred using neighbor-joining method in MEGA3.1 software.

Table S1. Expression and phenotype analysis of transgenic plants

Genotype	Relative Expression levels of exgenous genes (Phenotype)									Total number of transgenic plants
	1	2	3	4	5	6	7	8	9	
35S:GL1-SRDX	390(+)	289(+)	540(+)	123(++)	78(++)	178(++)	12(+++)	2(+++)	8(+++)	27(24)
35S:MYB82-SRDX	1200(+)	3078(+)	1535(+)	561(++)	328(++)	180(++)	10(+++)	15(+++)	23(+++)	32(24)
35S:nMYB82	864(+)	960(+)	1865(+)	235(++)	129(++)	98(++)	1(+++)	6(+++)	6(+++)	47(38)
35S:MYB82g	354(++)	769(++)	435(++)	1542(++)	932(++)	1876(++)	32(+++)	57(+++)	8(+++)	30(21)
35S:MYB82c	1209(+++)	12(+++)	543(+++)	879(+++)	23(+++)	187(+++)	2018(+++)	239(+++)	1209(+++)	37(0)

9 transgenic individuals were selected according to trichome phenotypes and used for gene expression analysis. "+" indicates that trichome number is similar to *gl1* mutant; "++" indicates that trichome number is less than wild type but more than *gl1* mutant, "+++" indicates that trichome number is similar to wild type. The number in parathesis indicates the number of transgenic plants with less trichomes than wild type.

Table S2. Primers used in this article.

qGL1-F: GGCACTGGCCAATGGAACCG
qGL1-R: GCCGAGGAGCTTGTGGAGACGA
qGL2-F: ACACGGGCGTCTTTGCCCTC
qGL2-R: ACGGCCAGTCTCAACGCTGC
qMYB82-F: TCGCTGGTCGCCTTCCAGGT
qMYB82-R: TGAAAGGAGTGGCGCCGACG
qACTIN2-F:TGTGCCAATCTACGAGGGTTT
qACTIN2-R:TTTCCCGCTCTGCTGTTGT

yhMYB82-F: TTTgaattcATGGAATGCAAAAGAGAAGAAGGG
yhMYB82-R: TTTggatccCTAAAGCAGAGGAAAGAAATCGGT
yhGL1-F: TTTgaattcATGAGAATAAGGAGAAGAGATGAA
yhGL1-R: TTTggatccCTAAAGGCAGTACTCAACATCAC
yhGL3-F: TTTgaattcATGGCTACCGGACAAAACAGAA
yhGL3-R: AAAggatccACAGATCCATGCAACCCTTTGA

MYB82-F: TTTgagctcATGGAATGCAAAAGAGAAGAAGGG
MYB82-R：TTTggatccCTAAAGCAGAGGAAAGAAATCGGT
nMYB82-R: AAAgtcgactcaTCCATGGCTTCTTCTCAGTTCTGT
myb82-SRDX-R: TTTggatccCTACAAACGGAGTTCTAGATCAAGCAGAGGAAAGAAATCGGTAAAG
GL1-SRDX-R: TTTggatccCTACAAACGGAGTTCTAGATCAAGGCAGTACTCAACATCACCA

ProMYB82-F: GATGCCTATGTGTGTGCAAGCTTTG
ProMYB82-R: TTgagcTCCACAGTTTTGCAAGTGAGTGAGG

amiR-myb82-F: TAGATATCCTAtaggctatgatgtccacgtatTGGGGATACAATTTTTCTAAATG
amiR-myb82-R: AAGAATTCCGGtaggctatgacgtccacgtaaTGGATAATCATTTAGAAAAATTG

GAL4-BD-F: AAAgagctcATGAAGCTACTGTCTTCTATCGAACA
GAL4-BD-R: AAAggatccCGATACAGTCAACTGTCTTTGACCT
GAL4-AD-F: TTTggatccATGGATAAAGCGGAATTAATTCC
GAL4-AD-R: AAAgtcgacCTCTTTTTTTGGGTTTGGTGG
